# Large language models and conditional rules in clinical decision support systems

**DOI:** 10.1007/s13755-026-00428-z

**Published:** 2026-01-21

**Authors:** Shangeetha Sivasothy, Adrian Bingham, Irini Logothetis, Scott Barnett, Mohamed Abdelrazek, Carl Luckhoff, Joseph Mathew, Rajesh Vasa, Kon Mouzakis

**Affiliations:** 1 Applied Artificial Intelligence Initiative, Deakin University, Geelong, VIC Australia; 2 Emergency and Trauma Centre, Alfred Health, Melbourne, VIC Australia; 3 National Trauma Research Institute, Alfred Health, Melbourne, VIC Australia

**Keywords:** Clinical decision support systems, Conditional rules, Large language models

## Abstract

**Background:**

Clinical Decision Support Systems (CDSS) improve patient outcomes and support sustainable health services by enhancing medical decisions. Developing rules for a CDSS is expensive due to delays in capturing and defining the rules through multiple iterations between clinicians and developers as the role of a clinician is patient care.

**Objective:**

We investigate the effectiveness of large language models (LLMs) and large reasoning models (LRMs) in generating a triaging rule set for a CDSS.

**Methods:**

We prompt various LLMs (GPT-3.5, GPT-4, GPT-4o, Gemini, Claude 3.5 Sonnet) and various LRMs (GPT-o1-mini, Grok-4, Claude 4 Sonnet) using alternative prompting techniques. We compare the LLM generated rule sets against the clinical rule set from our Pandemic Intervention Monitoring System (PiMS); a triaging CDSS built in collaboration with clinicians to monitor COVID-19 positive patients. Effectiveness is evaluated based on the accuracy, interpretability, and rule complexity.

**Results:**

We identified that LLMs generated COVID-19 screening rule sets compared to triaging rule sets when not specifying the variables from our PiMS rule set. By including PiMS variables in our prompts, we discovered LLMs 1) had lower interpretability and rule complexity compared to the PiMS rule set, and 2) resulted in an average accuracy between 31.62% ± 0.19% and 70.71% ± 0.02%. While for LRMs, we identified that 1) interpretability varied between 3 and 94 compared to 41 identified in our PiMS rule set and 2) resulted in an average accuracy between 31.62% ± 0.19% and 81.70 ± 0.05%.

**Conclusions:**

LLMs are limited in emulating clinical rule sets due to their simplicity and lack of complex reasoning. Despite LRMs improving effectiveness, they are still limited. LLMs and LRMs can generate a feasible initial rule set for CDSS. This can reduce time invested by clinicians and developers by minimising the number of iterations for refinement. Future work can explore integrating LLMs and LRMs with decision trees to improve effectiveness.

**Supplementary Information:**

The online version contains supplementary material available at 10.1007/s13755-026-00428-z.

## Introduction

Clinical Decision Support Systems (CDSS) are interactive systems whereby clinicians enter data that is parsed through a knowledge base returning a system recommendation supporting clinical decisions [1]. These systems collect large amounts of patient data and process it efficiently to extract insights based on embedded clinical knowledge and provide recommendations to enhance clinical decision-making. CDSS can encapsulate clinical knowledge in the form of rules providing capability for data analysis in real-time providing more efficient and effective care supporting sustainable health services [2].

**Fig. 1:**
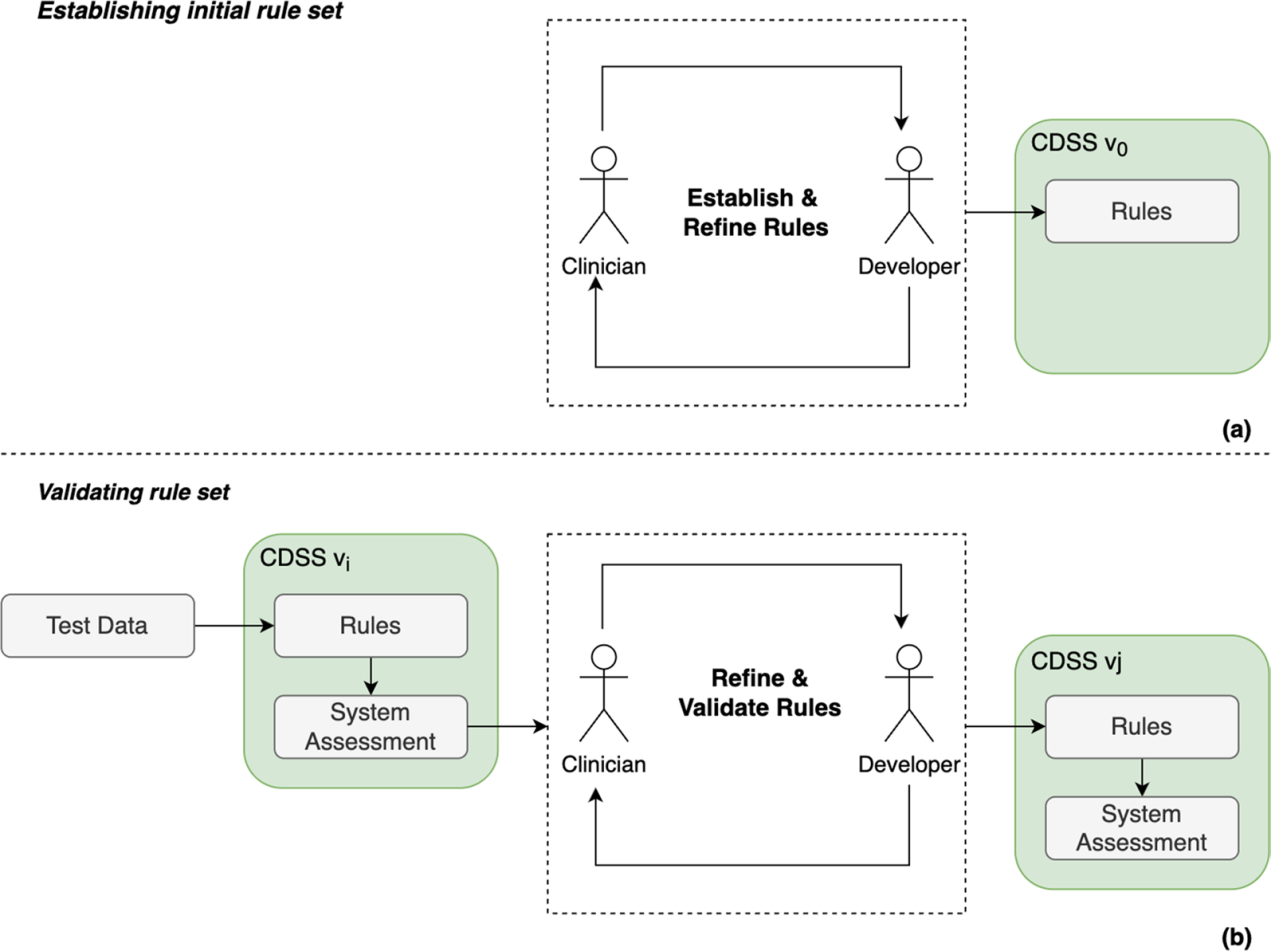
High level process depicting the collaboration between clinicians and developers to create rule sets for CDSS through multiple iterations: **a** the iterative process for establishing and refining an initial rule set, **b** the iterative process for refining and validating rules through an iterative process resulting in multiple CDSS versions

To develop tailored rule sets for a CDSS, clinicians collaborate with developers to capture clinical knowledge into a rule set that guide decision-making. This phase of development includes three stages: (i) knowledge extraction into rules [3], (ii) evaluation of rules [3–7], and (iii) validation of rules in clinical settings [8–10]. Clinicians are needed across these three stages resulting in a high demand on their time. This is exacerbated as it involves continuous refinement through an iterative approach at each stage (i-iii). However, a clinician’s priority is duty of care [11], and due to limited staffing resources, the availability of clinicians to support knowledge base development is limited [12]. Figure 1 is a high-level representation of the engagement between a clinician and developer through multiple iterations to establish and refine rules for the CDSS. The nuances of this engagement are outside the scope of this work.

Previous works have indicated that rules can be extracted from clinical practice guidelines, publications, multimedia resources, and internet resources [13, 14]. Clinical practice guidelines vary between different medical organizations and become outdated. Many publications are behind paywalls limiting access. Given the vast number of publications, it is challenging to stay fully informed on the latest findings. Multimedia resources and internet resources are high in volume, making it difficult to filter relevant information. In contrast, Large Language Models (LLM) provide a centralised source of information by processing and integrating data from a wide range of sources. We aim to investigate LLMs as an alternative source for generating an initial rule set for clinicians to modify and adjust, allowing clinicians to be more efficient during the development phase.

Current research on automated rule generation for CDSS focus on established AI techniques, such as machine learning and deep learning. However, these approaches are effective only when large datasets with minimal sparsity (variability) are available. This was not feasible during the pandemic, when no data was available for our rule set [15]. In addition, high sparsity is common in general medical datasets, such as those from emergency and trauma care [16, 17]. Thus, human engineered rules remain a common approach.

Prior research presents capabilities of LLMs in healthcare for knowledge retrieval [18, 19], synthesis [20], to extract rules[21], and providing recommendations [22, 23]. They have been used for diagnosis [3–5, 24], prognosis [25, 26], and patient monitoring [27] extending to clinical knowledge question and answering tasks [28–32]. Previous studies [33–36] have demonstrated the application of LLMs within CDSS in assisting decision-making tasks, such as medical image analysis for diagnosis, report generation, and medication management. Also, these studies [33–35] have used LLMs to answer questions from clinicians. However, previous research has not leveraged LLMs and Large Reasoning Models (LRM) for clinical rule extraction, which is crucial for developing CDSS.

He et al. [21] and Kim et al. [37] applied generative models for rule extraction from text and standard medical protocols and classifications. However, this research is only practical for medical conditions proven in medical practice. Previous research has not leveraged LLMs for clinical rule generation, our research is crucial for developing CDSS when there is limited to no data or information. In addition, our research is evaluated relative to a benchmark whereby we compare the LLM and LRM generated rule set to a proprietary clinically validated and trialled rule set [38].

**Fig. 2:**
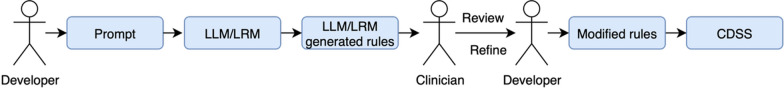
The proposed approach leveraging LLMs or LRMs reduces iterations between clinician and developer whereby the developer generates an initial set of rules using LLMs or LRMs and then clinicians can review and refine these LLM or LRMs prior to the developer embedding them into the CDSS

Leveraging LLMs to generate conditional rules for a CDSS knowledge base creates the foundation for streamlining the knowledge extraction stage (stage i) enabling clinicians to focus on the evaluation and validation stages (stages ii-iii). Increasing the efficiency and effectiveness of knowledge extraction can result in less iterations for evaluating and validating the rule sets; thus, reducing the time commitment from clinicians and time invested by developers. Figure 2 illustrates how the introduction of LLMs reduces the number of iterations between clinicians and developers.

In our study, we aim to evaluate the effectiveness of LLMs and LRMs in generating rules for a CDSS, specifically a COVID-19 patient triaging system. We conducted a comparative analysis on their performance against a predefined rule set created for a COVID-19 patient triaging system in production, Pandemic intervention Monitoring System (PiMS). This predefined rule set was created by senior emergency, trauma and infectious disease clinicians with over 80 years combined experience.

To conduct this study, we evaluated the three most common and popular LLMs: ChatGPT (GPT-3.5, GPT-4, GPT-4o), Gemini, and Claude 3.5 Sonnet [4, 39–41]; and the three most common and popular large reasoning models (LRMs) ChatGPT (GPT-o1-mini), Grok-4, and Claude 4 Sonnet. These three LLMs and three LRMs were selected due to their distinctive capabilities in reasoning, context understanding, or medical domain knowledge. We applied six common prompting techniques on the selected LLMs and LRMs to generate the rule sets for evaluation. We measured the effectiveness of LLM and LRM generated rules in terms of accuracy, interpretability, and rule complexity. For accuracy, we applied the health data from PiMS to the rule set generated by the LLMs and LRMs to assign triaging levels. These triaging levels were then compared to those assigned by the PiMS system, and accuracy was calculated as the percentage of data points correctly triaged by the LLM and LRM rule set compared to the clinical PiMS rule set. Interpretability is a measure of the number of conditional rules generated in the rule set [42, 43]. Conditional rules consist of two parts a Boolean condition (true/false) and a result. Rule complexity is a measure of the number of different paths dependent on the conditions when executed [44]. A greater rule complexity implies an increase in paths to reach an assessment that can lead to a low accuracy compared to the PiMS rule set.

Our first contribution establishes a methodology to evaluate effectiveness of LLMs and LRMs and prompting techniques in generating rules for CDSS. This methodology is generalisable and can be used for different rule sets across various LLMs and LRMs. Our second contribution demonstrates that different prompting techniques yield different effectiveness relative to the LLM and LRM in the context of health. Our research emphasises the need for repeatability in LLM and LRM studies.

## Background

### Pandemic intervention monitoring system (PiMS)

The CDSS used for this study was the Pandemic Intervention Monitoring System (PiMS) [38]. PiMS was developed in collaboration with clinicians during the COVID-19 pandemic to assist clinicians in classifying the status of COVID-19 positive patients. The rule set was manually developed by emergency, trauma and infectious disease clinicians with over 80 years’ experience and validated by deploying it in two hospitals. PiMS was selected as we have access to its: (1) dataset to evaluate accuracy and (2) rule set to evaluate interpretability and rule complexity [38].

The PiMS dataset comprises records of 109 COVID-19 positive patients. Patients were requested to submit their health data, specifically vitals and symptoms. This patient data was parsed through the rule set and a system assessment was conducted assigning a triaging level to the patient’s health data. The triaging levels include red, amber, green, and uncertain. Amber was assigned by default and PiMS applied the rule set to the health data for red, green and uncertain overriding the default triaging level if applicable. Red and green define high risk and low risk patients respectively while uncertain is assigned for missing or incomplete health data. Dedicated nurses reviewed the patient’s health data validating or overriding the system assessment.

Patients submitted their health data 3 times a day by default and 5 times a day for patients at risk as determined by the rule set. The duration for patient health data collection ranged from 7 to 18 days. This resulted in a dataset with 3277 triaging levels from the system assessments based on the clinical rule set.

### Large language models and large reasoning models

Large language models (LLMs) are deep learning neural networks built on a transformer architecture that contain tens to hundreds of billions of parameters, and pre-trained on 400 TB of uncompressed data (~ 3 billion web pages) for natural language processing tasks. Our study focuses on three LLMs (five versions) under extensive investigation in recent research, including ChatGPT (GPT-3.5, GPT-4, GPT-4o), Gemini, and Claude 3.5 Sonnet [4, 39–41]. GPT-3.5, GPT-4 and GPT-4o leverage a decoder only transformer architecture developed by OpenAI. Google’s Gemini uses a multimodal architecture built on transformer decoders that is optimised for efficient and reliable performance at scale. Claude 3.5 Sonnet developed by Anthropic, focuses on safe and ethical AI whilst maintaining high performance in language understanding and generation. GPT-3.5, Gemini and Claude 3.5 Sonnet are free to use.

Large Reasoning models (LRMs) are LLMs that encompass prompting techniques, reasoning techniques and reinforcement learning to conduct reasoning. LRMs incorporate an internal chain of thought prior to producing an output. LRMs are known to perform well in complex problem solving, coding, scientific reasoning, and multi-step planning for agentic workflows. Our study focuses on three reasoning models common in recent research, including ChatGPT (GPT-o1-mini), Grok-4, and Claude 4 Sonnet. GPT-o1-mini is a LRM developed by OpenAI and is tailored for STEM tasks such as coding and math. Grok-4 is developed by xAI is the largest model to date while Claude 4 Sonnet developed by Anthropic, has maintained its focus on safe and ethical AI. Grok-4 and Claude 4 Sonnet are free to use.

### Prompting techniques

Prompts are input queries given to LLMs and LRMs, directing them to generate relevant responses. Prompting involves designing and refining prompts to elicit desired responses from LLMs and LRMs. We have selected prompting techniques that have been extensively investigated in recent research, including: i) Role-play, ii) Instruction Following, iii) Chain of Thought (CoT), iv) Few-Shot, v) Few-Shot + Chain of Thought (Few-Shot + CoT) and vi) Sequential.

Role-play prompting directs the LLM and LRMs to ‘imitate’ and respond as a human [45]. For example, “you are a clinician” would direct the LLM and LRMs to respond from a clinician’s perspective. Instruction Following involves providing the LLM and LRMs explicit instructions to complete tasks, with all necessary details outlined in the prompt [46]. Extending Instruction Following, CoT prompting guides the LLM and LRMs through a sequential reasoning process [39]. Phrases such as “let’s think step by step” [47] encourages the LLM and LRMs to tackle tasks by performing and presenting its logical deductions. Instruction Following directs the LLM and LRMs to perform a specific task through instructions whereas CoT prompting encourages step by step reasoning and produces intermediate outputs before arriving at the final output. Few-Shot prompting improves performance by including examples of an expected structural response from the LLM and LRMs in the prompt [47] influencing the model’s response. Few-Shot + CoT prompting combines Few-Shot and Chain of Thought prompting where the prompt includes examples of an expected response and a phrase to conduct and present its deductive reasoning[39]. Sequential prompting uses a series of prompts in a sequence, each building on the previous one by referring to its output; thus, guiding the LLM and LRMs step by step [48].

## Methodology

We have selected role-play prompting due to its ability to mimic clinicians (subject-matter experts) [45], instruction following because it offers step-by-step guidance [46], chain of thought prompting for its reasoning capacity [39], few-shot prompting for its iterative nature and refinement based on examples [47], few-shot + CoT prompting because it offers multi-step reasoning [39], and sequential prompting because it provides a series of interrelated prompts to guide the LLM and LRM step-by-step [48]. For the few-shot prompting technique, we provided a non-medical example, specifically from finance to eliminate prompting using domain specific terminology.

### Study design

**Fig. 3:**
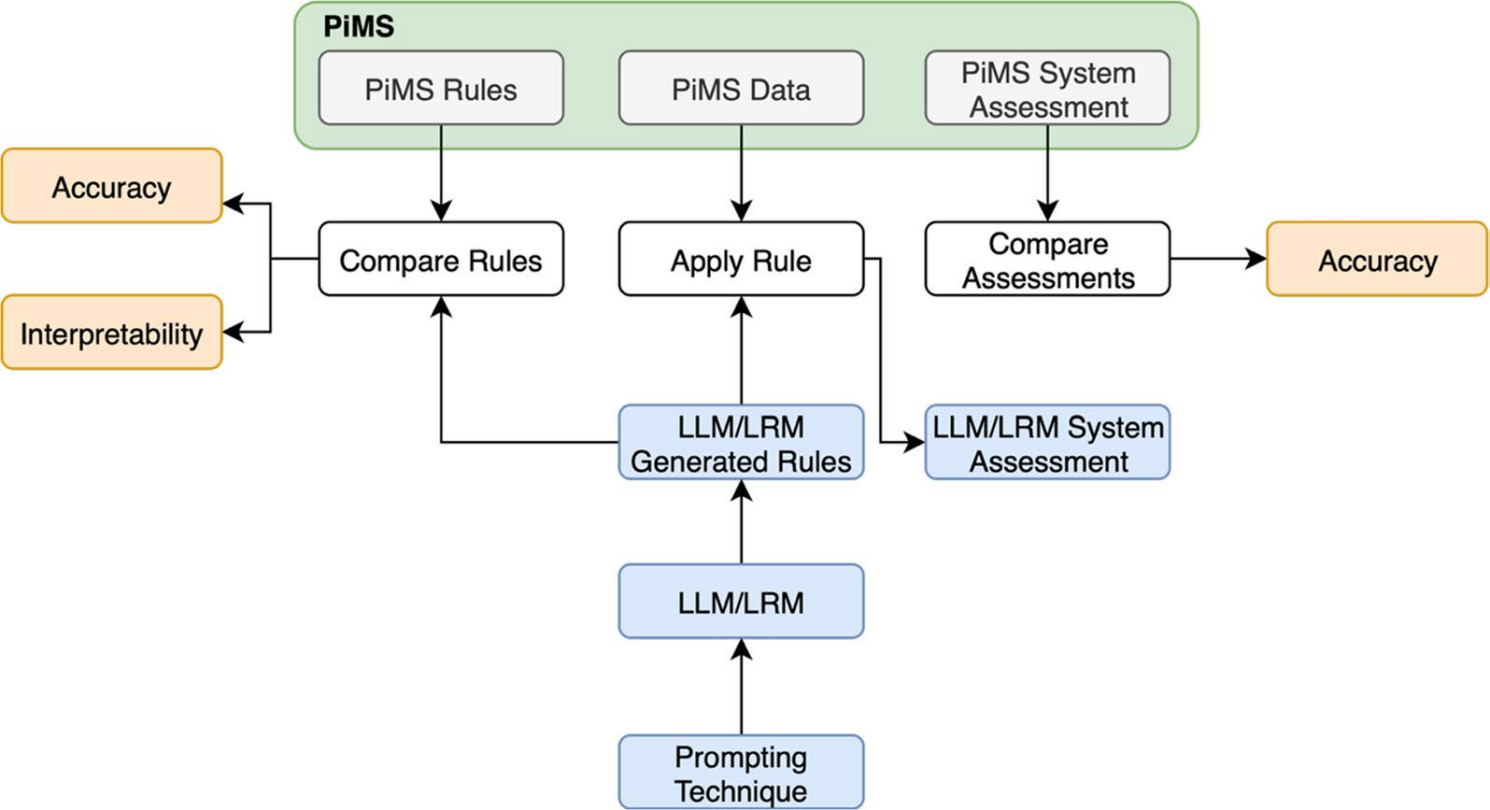
Our research methodology for evaluating accuracy, interpretability and rule complexity of LLM and LRM generated rules against PiMS rules

To evaluate the effectiveness of the selected LLMs and LRMs for generating rule sets for a COVID-19 patient triaging CDSS, we conducted a comparative analysis of the generated rules against our PiMS clinical rule set. Figure 3 presents the overall research methodology, comparing LLM generated rules with PiMS rules in terms of interpretability and rule complexity, and evaluating LLM and LRM system assessments against PiMS system assessments in terms of accuracy. This study was conducted in two parts, Part A applied role-play, instruction following, CoT, and few-shot prompting techniques for the LLMs to generate a rule set without defining any variables in the prompts. These prompting techniques were assessed on common LLMs, such as GPT-3.5 and GPT-4. From this assessment, we refined our prompts for Part B and extended our experiments to include GPT-4o, Gemini and Claude and LRMs GPT-o1-mini, Grok-4 and Claude 4 Sonnet. For Part B, we extended Part A by (i) including few-Shot + CoT and sequential prompting techniques, (ii) defining the variables and associated scales in the prompts as per the PiMS rule set. During sequential prompting, we asked the LLM and LRM to generate a clinical protocol, then outline rules for daily monitoring, and finally produce Python code to implement those rules, ensuring each response built upon the previous ones. Both Part A and Part B were executed using the LLM web interfaces and LRMs using an API key for GPT-o1-mini and web interfaces for Grok-4 and Claude 4 Sonnet.

Table 1 presents the prompting technique and prompts used in Part A including the type of prompt, such as a single prompt or a sequence of consecutive of prompts. We have not presented prompts used in Part B due to IP protection. Given that our PiMS rule set is in Python, our prompts included instructions for the LLM and LRM to generate the rule set in Python to expedite the comparative analysis. We repeated the experiment ten times to account for the non-deterministic nature of LLMs and LRMs.

**Table 1:** Different prompting techniques and used prompts during the experiments

Technique	Type	Prompt Part A
Role-play [45]	Single	System prompt: You are a clinician User prompt: Write COVID-19 pandemic intervention monitoring rules in Python code
Instruction Following [46]	Single	Write COVID-19 pandemic intervention monitoring rules in Python code
CoT [39]	Consecutive	Prompt 1: Write 25 keywords for pandemic intervention monitoring Prompt 2: Write 15 rules using these keywords Prompt 3: Out of these rules, which can be implemented using conditions such as greater than Prompt 4: Write precise and concise rules in Python code
Few-shot [47]	Single	You are subject matter expert (SME). You are helping a software development team to build software for your domain. You do this by building rule sets that use variables to determine actions/outcomes. Rule set formatting: """ You also provide definitions for all variables, and constants. The variables used must be collected by digital systems For example: Problem domain: Financial services Objective: Fraud detection Rule 1: IF TransactionAmount > $1000 AND TransactionCountry ! = UserHomeCountry THEN “potential fraud” Rule 2: IF NumberOfOriginCountriesInLastTwoMinutes > 1 THEN “potential fraud” Rule 3: IF TransactionAmount > $1000 AND TargetAccountCountry ! = UserHomeCountry THEN “potential fraud”...etc Variables: TransactionAmount = Current transaction amount in $ TransactionCountry = Where a transaction was made NumberOfOriginCountriesInLastTwoMinutes = Number of unique countries transactions were sourced from in the last two minutes. UserHomeCountry = The country the current user originates from. """ Problem domain: Medical Objective: Classify COVID-19 patients health status to help clinicians determine if they should receive medical attention. The classification must be as follows: GREEN, AMBER, RED

### Configuration of LLMs and LRMs

For the LLMs, we configured their temperature and maximum response token. Temperature controls the randomness of the model’s output by changing variability in responses while maximum response token length sets a limitation on the length of the output. For GPT-3.5, GPT-4, and GPT-4o, we set the temperature to 0 guiding the LLM to respond with the highest-probability output reducing its non-deterministic nature. This configuration is set to 1 by default for Gemini and Claude 3.5 Sonnet and cannot be changed through the web interface. We set the maximum response token length to 3000 for GPT-3.5, GPT-4, and GPT-4o, while for Gemini and Claude 3.5 Sonnet, we used their default maximum response token lengths of 8192 and 4092, respectively. For the LRMs, we configured their maximum response token settings to a length to 5000 for GPT-o1-mini, while for Grok-4 and Claude 4 Sonnet, we used their default maximum response token lengths of 8000 and 128,000, respectively. For the LRMs, temperature is not required to be configured as its effect is negligible.

### Evaluation

To assess the quality of the rule sets generated by LLMs and LRMs, we evaluated their accuracy, interpretability, and rule complexity compared to the clinical rule set currently used in PiMS.

Accuracy was assessed by applying patient data collected from PiMS to the rules generated by the LLMs and LRMs. Each patient's data was evaluated using both the LLM/LRM-generated rules and the existing clinical rules in PiMS, resulting in two separate triage level assignments. The accuracy of the LLM/LRM-generated rule set was then determined by calculating the percentage of cases in which the LLM/LRM’s triage matched the PiMS clinical triage.

Interpretability was evaluated based on the simplicity of the rules, specifically by counting the number of conditions within each rule set. Each conditional rule could include one or multiple criteria; simpler rules with fewer conditions were considered easier to interpret and apply clinically. Interpretability is measured by counting number of rule blocks inside the entire code snippet.

Complexity was assessed by examining the intricacy of each rule set, specifically counting the number of criteria or decision points needed to arrive at a triage decision. This complexity influences the simplicity of understanding the rules and reliability of implementation and applications in clinical practice. For example, decision points might include conditions such as “patient temperature greater than 38C,” “respiratory rate exceeding 20 bpm,” or more nuanced criteria such as “recent travel history to regions with known disease outbreaks.” Complexity is a measure of the keywords where decisions are made during the execution path. These keywords include IF, AND, OR, ELSE IF, ELSE.

Rule sets with fewer decision points might not adequately capture important clinical considerations, potentially overlooking critical patient needs. Conversely, rule sets with numerous decision points might include redundant or irrelevant criteria, such as unnecessarily detailed patient history information that does not directly impact immediate triage outcomes, making them overly complicated and difficult to apply accurately.

We randomly reviewed 70 LLM/LRM-generated rule sets to identify rule sets that contained either overly complex or insufficient rules compared to the clinician-defined rules in PiMS. For LLM/LRMs rule sets that had significantly more decision points, this indicated unnecessary complexity, suggesting the inclusion of rules that did not meaningfully enhance clinical decisions. For rule sets that had significantly fewer decision points, this indicated a possible omission of critical clinical criteria essential for accurate patient triage. In addition, we assessed logical errors. Logical errors are errors relative to reasoning and typically involves an initial assessment that is accurate being overridden due to an error in the logical structure of the rule set. Logical errors are common in edge cases.

However, even when LLM/LRM-generated and clinician-designed rule sets had a similar number of decision points, it did not necessarily imply that both sets covered the same clinical scenarios. Clinicians typically prioritize rules to handle rare or clinically critical situations (edge cases) [49], such as rare allergic reactions or uncommon disease presentations, whereas LLMs and LRMs generally emphasize patterns observed more frequently in the data [50]. Determining precisely which rules are essential or redundant is beyond the scope of this current evaluation.

To measure complexity quantitatively, we used rule complexity [44] as our metric. Rule complexity quantifies the complexity of a rule set by counting the number of independent decision pathways or conditions within it. Our aim was to align the LLM/LRM-generated rule sets as closely as possible with the clinically established rule set.

***Ethical and IP Considerations.***

PiMS received ethics approval from Alfred Health [208/20]. This approval ensures that all data usage complies with the ethical standards. Due to this governance, we did not include any PiMS data into the LLM and LRM prompts. We cannot share the PiMS rule set due to IP protection.

## Results

### Part A

Table 2 presents the accuracy of LLM assessments for the experimental iterations of the various LLMs that generated rule sets that included variables comparable to our PiMS rule set. We limited our evaluation to few-shot prompting in Part A given that the variables in the generated rule sets were comparable to PiMS. Other prompting techniques specified rules using variables which were not included in our PiMS rule set. From the 20 experimental iterations (10 iterations from GPT-3.5 and 10 iterations from GPT-4 using few-shot prompting), only 6 iterations included rule sets with variables comparable to the PiMS rule set variables. The remaining 14 iterations included variables that were not defined in the PiMS rule set. When executing the LLM rule set on the PiMS patient data and comparing system assessments of the LLM generated rules and PiMS rules, we received an accuracy varying between 1.7 and 26.4%. The former was achieved by GPT-3.5 in iteration 10 whilst the latter was achieved by GPT-3.5 in iteration 6. GPT-4 reported accuracy of 17.5% and GPT-3.5 reported accuracies of 18.1, 17.2, 26.4, 23.5, and 1.7%.

**Table 2:** Accuracy of LLM assessments for the experimental iterations whereby the variables included in the generated rules were comparable to the PiMS variables

Model	Iteration Number	Accuracy
GPT-3.5	2	18.1%
GPT-3.5	3	17.2%
GPT-3.5	6	26.4%
GPT-3.5	9	23.5%
GPT-3.5	10	1.7%
GPT-4	2	17.5%

Table 3 compares the domain-specific variables from the rule sets generated by GPT-3.5 and GPT-4 across 10 experimental iterations to those found in the PiMS rule set. GPT-3.5 and GPT 4 generated rule sets that included only 33% and 47% of the variables used in our PiMS rule set, respectively. Both GPT-3.5 and GPT-4 failed to include two common COVID-19 symptoms, sore throat and runny nose. Despite GPT-4 including comorbidities such as hypertension, chronic respiratory disease and diabetes, it did not include cardiac disease and immunosuppressed. Thus, comorbidity was not included in the variable count as we cannot conduct a comparison.

**Table 3:** Comparison of domain-specific variables on LLM generated rule sets and PiMS rule set without defining variables in our few-shot prompts

Variable	GPT-3.5	GPT-4	PiMS
Body Temperature	x	x	x
Shortness of Breath		x	x
Cough	x	x	x
Loss of Taste or Smell		x	x
Sore Throat			x
Respiratory Rate	x		x
Fatigue	x	x	x
Oxygen Saturation		x	x
Heart Rate	x		x
Age		x	x
Comorbidity			x
Gender			x
Myalgia			x
Diarrhoea			x
Runny Nose			x
	33%	47%	100%

Assessing the interpretability, PiMS had a rule set of 41 whereby GPT-3.5 and GPT-4 scored low ranging between 3–4 and 2–4, respectively, across the 10 experimental iterations. This shows that LLMs generate fewer rule sets compared to PiMS that provides comprehensive and detailed rule sets.

Our PiMS rule set had a rule complexity of 44. The rule complexity of rule sets generated using few-shot prompting technique by GPT-3.5 varied between 5 and 7 with a median of 7. The rule complexity of rules generated using few-shot prompting technique by GPT-4 varied between 3 and 14 with a median value of 8. The low rule complexity of our LLM generated rule set indicates the LLM missed rules. Given the rule complexity is a measure of linear paths through the rule set, a lower value indicates fewer paths compared to the PiMS rule set. Thus, the LLM generated rule set has less complexity compared to the PiMS rule set.

### Part B

Table 4 presents the average accuracy and standard deviation from the ten iterations when we included the PiMS variables in our prompts, the highest accuracies are in bold. The highest accuracies for GPT-3.5, GPT-4, GPT-4o, Gemini, and Claude 3.5 Sonnet were 86.42, 74.21, 84.83, 76.99, and 84.86%, respectively. The lowest accuracies for GPT-3.5, GPT-4, GPT-4o, Gemini, and Claude 3.5 Sonnet were 11.20, 19.22, 1.80, 1.30, and 21.85%, respectively. Overall, GPT-3.5 resulted in the highest accuracy when using the sequential prompting technique, scoring 86.42% The highest accuracy for GPT-4o and Claude 3.5 Sonnet was through chain of thought prompting, scoring 84.83% and 84.86% respectively. The accuracies for Claude 3.5 Sonnet across all prompts were above 20%. GPT-4o and Gemini resulted in accuracies below 10%, with some as low as 1–3%. Table 5 presents the average accuracy and standard deviation from the ten iterations when including the PiMS variables in our prompts, the highest accuracies are in bold. The highest accuracies for GPT-o1-mini, Grok-4, and Claude 4 Sonnet were 86.57, 89.47, and 88.07%, respectively. The lowest accuracies for GPT-o1-mini, Grok-4 and Claude 4 Sonnet were 4.49, 11.90, and 2.30%, respectively.

**Table 4:** Average accuracy and standard deviation of LLM system assessments to PiMS system assessments

Prompting Technique	GPT-3.5	GPT-4	GPT-4o	Gemini	Claude 3.5 Sonnet
Role-play	45.30% ± 0.06%	48.19% ± 0.13%	48.90% ± 0.06%	31.62% ± 0.19%	48.35% ± 0.15%
Instruction following	38.37% ± 0.08%	43.18% ± 0.02%	32.54% ± 0.16%	42.84% ± 0.27%	61.30% ± 0.17%
Chain of thought	56.04% ± 0.15%	43.66% ± 0.13%	49.10% ± 0.16%	45.33% ± 0.26%	**66.62% ± 0.21%**
Few-shot	42.90% ± 0.23%	45.53% ± 0.14%	32.02% ± 0.14%	**70.71% ± 0.02%**	46.90% ± 0.08%
Few-shot + Chain of thought	58.44% ± 0.01%	49.96% ± 0.13%	**52.78% ± 0.13%**	49.38% ± 0.18%	49.25% ± 0.06%
Sequential	**65.71% ± 0.23%**	**53.15% ± 0.10%**	58.24% ± 0.12%	42.71% ± 0.23%	53.02% ± 0.12%

The highest mean accuracies are in bold

**Table 5:** Average accuracy and standard deviation of LRM system assessments to PiMS system assessments

Prompting Technique	GPT-o1-mini	GPT-o1-mini	Claude 4 Sonnet
Role-play	54.58% ± 0.24	68.69% ± 0.21	57.90% ± 0.33
Instruction following	50.26% ± 0.20	67.36% ± 0.19	**70.93% ± 0.21**
Chain of thought	58.16% ± 0.25	36.28% ± 0.27	53.38% ± 0.32
Few-shot	44.67% ± 0.10	53.08% ± 0.10	69.40% ± 0.10
Few-shot + Chain of thought	55.67% ± 0.20	49.04% ± 0.15	59.75% ± 0.16
Sequential	**62.91% ± 0.09**	**81.70% ± 0.05**	59.04% ± 0.27

The highest mean accuracies are in bold

**Fig. 4:**
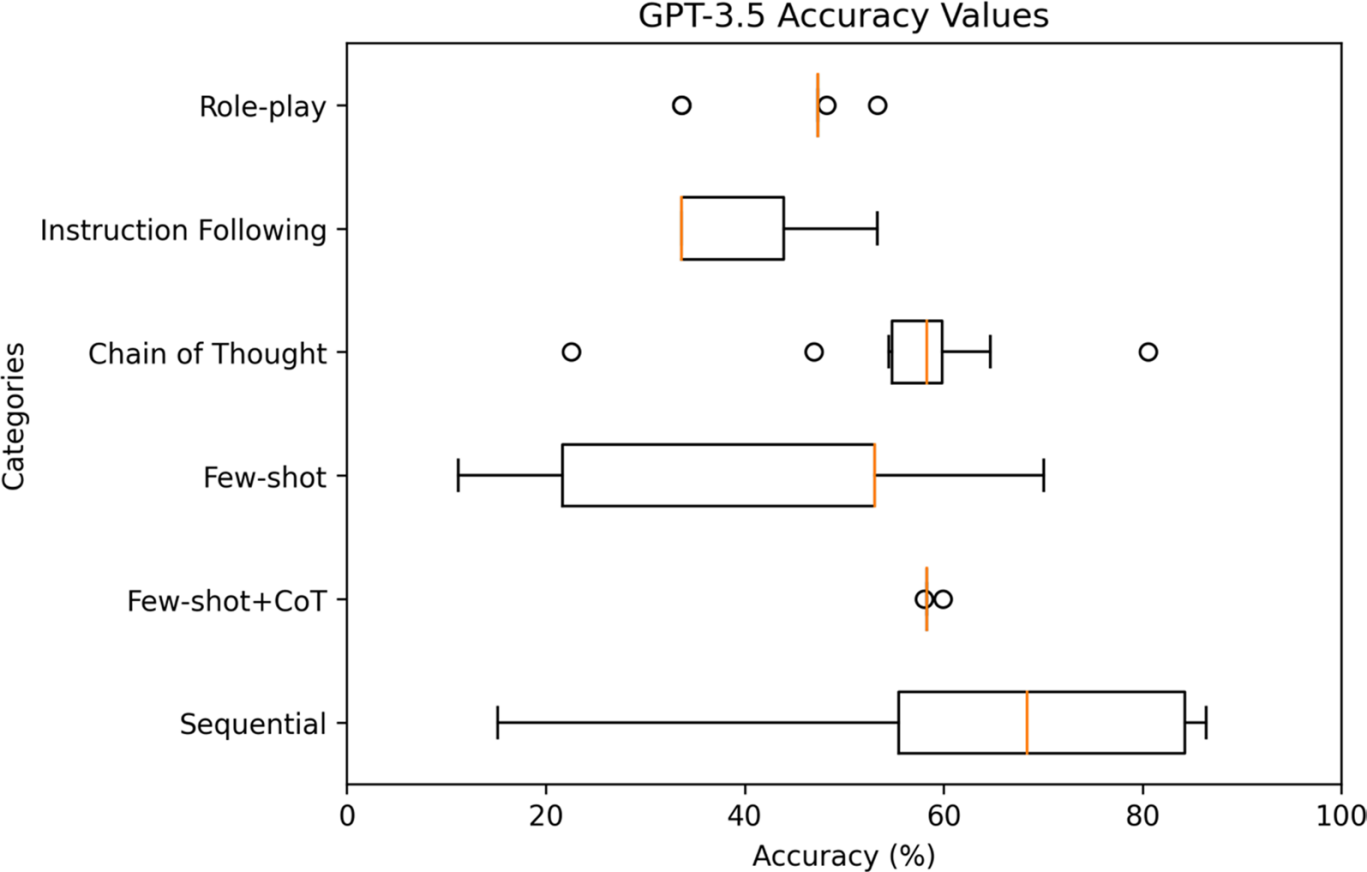
Boxplot showing accuracies when six different prompting techniques were executed on GPT-3.5 model

**Fig. 5:**
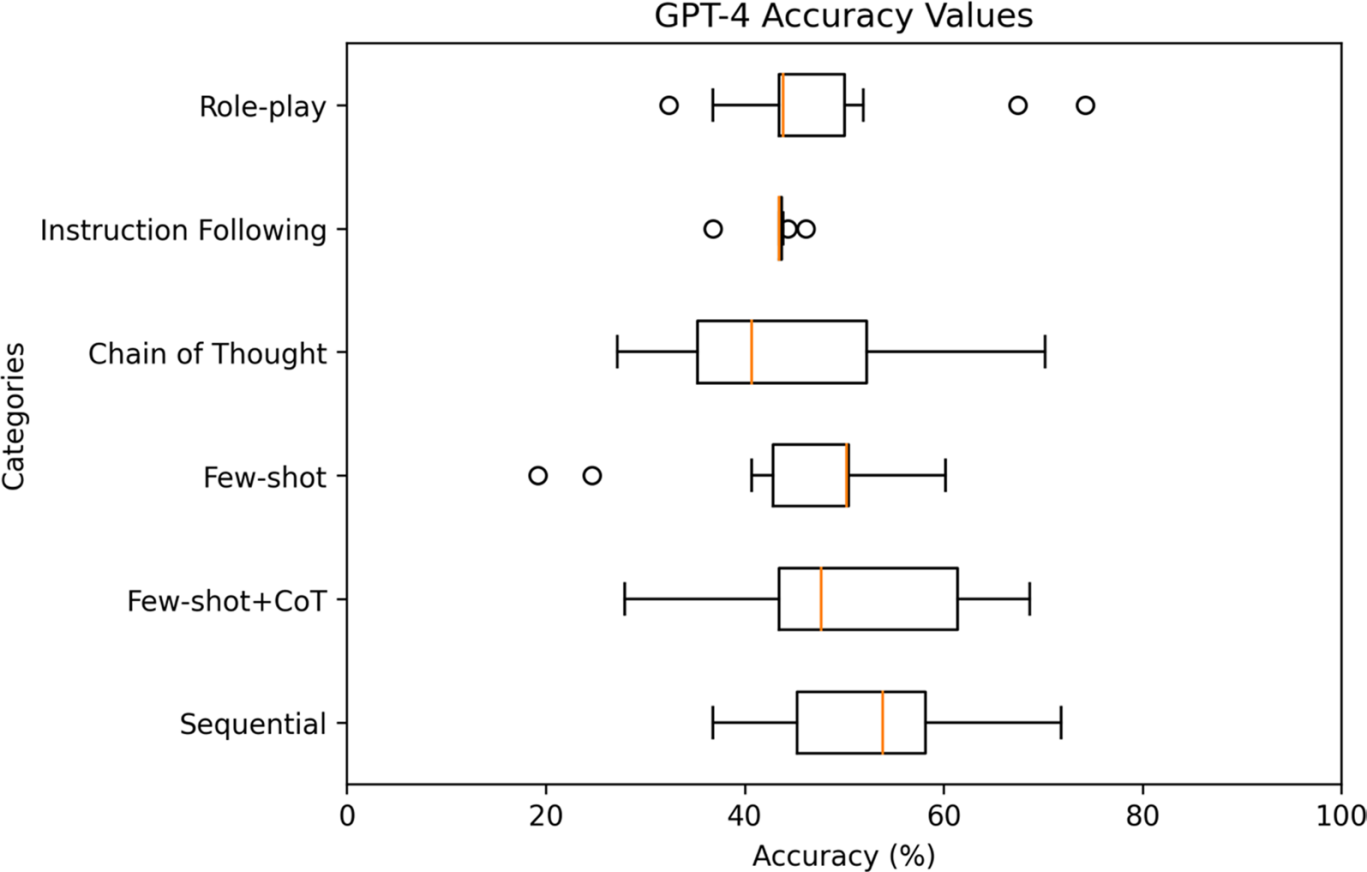
Box plot showing accuracies when six different prompting techniques were executed on GPT-4 model

**Fig. 6:**
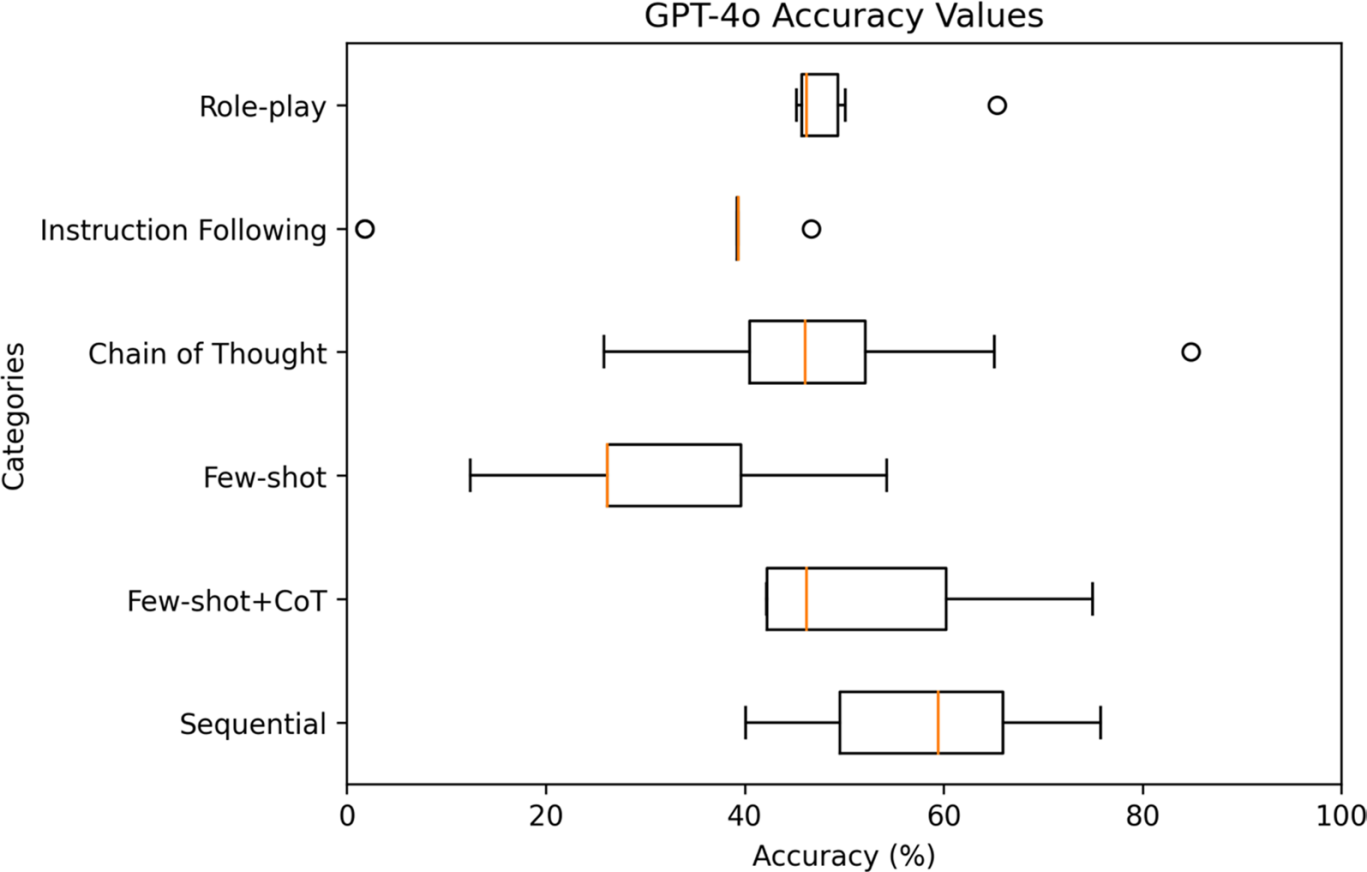
Box plot showing accuracies when six different prompting techniques were executed on GPT-4o model

**Fig. 7:**
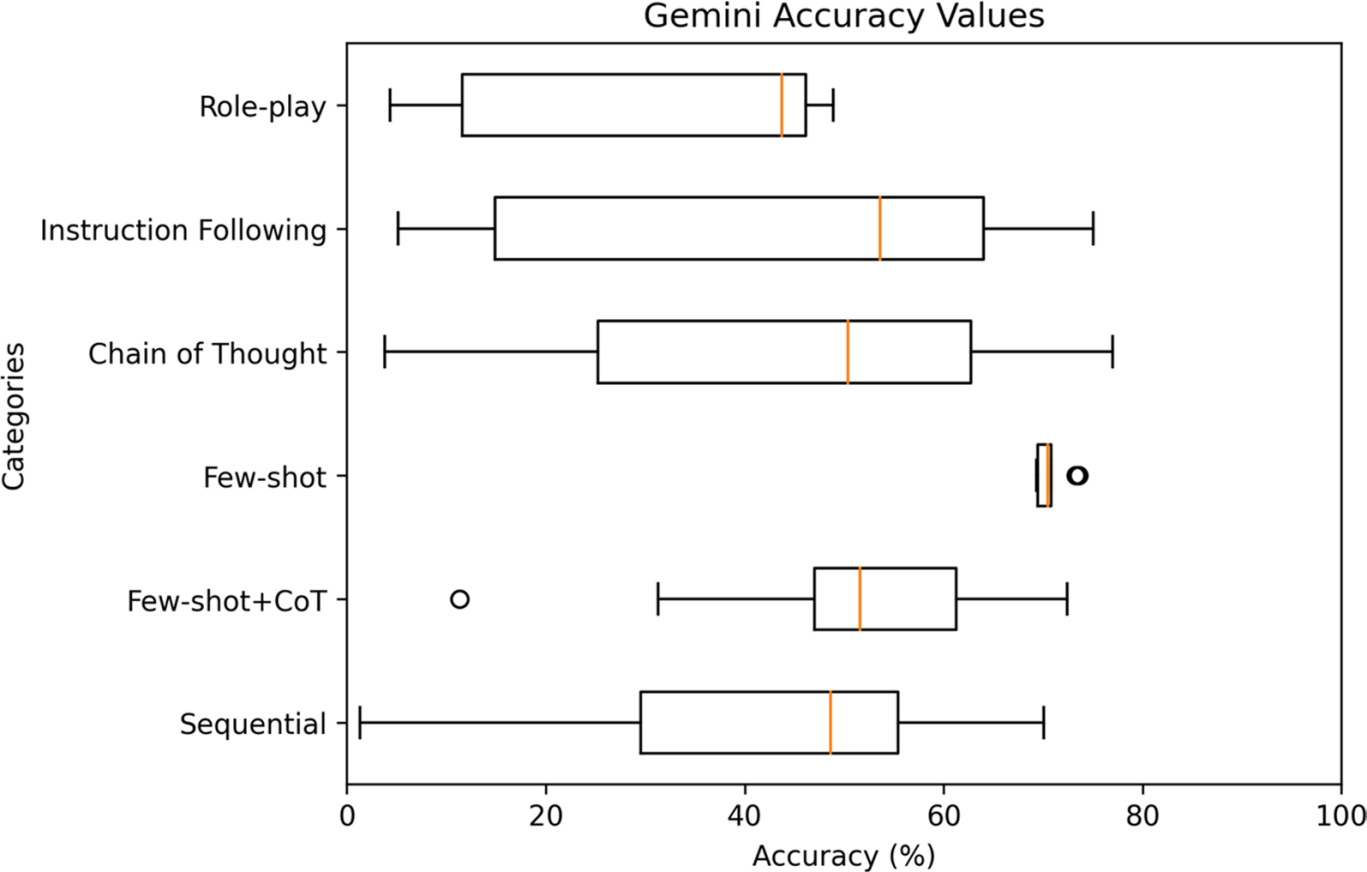
Box plot showing accuracies when six different prompting techniques were executed on Gemini model

**Fig. 8:**
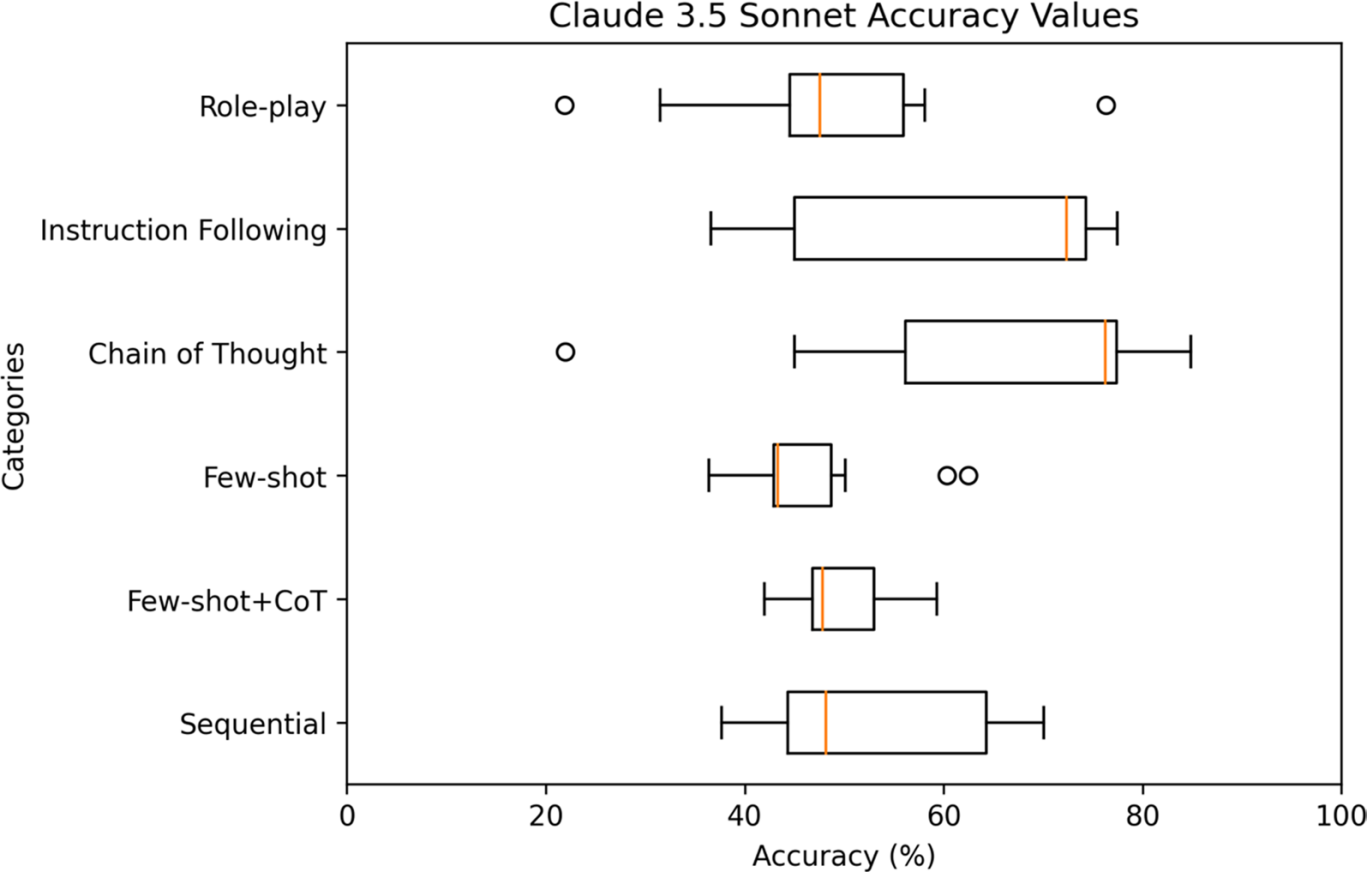
Box plot showing accuracies when six different prompting techniques were executed on Claude 3.5 Sonnet model

For a comprehensive analysis, Figs. 4, 5, 6, 7, 8 highlight the varying levels of accuracy across different prompting techniques on LLMs. For GPT-3.5 generated rules, few-shot + CoT and role-play had minimal impact on the accuracy resulting in low variability (58.04– 59.93%), refer to Fig. 4 In contrast, sequential and few-shot demonstrated significant impact in accuracies compared to other techniques, resulting in high variability (15.17– 86.42% for sequential and 11.20– 70.03% for few-shot). This was evident in GPT3-5 having the highest accuracy (86.42%) through sequential prompting technique. For GPT-4, instruction following showed less variability (36.77–46.14%) compared to chain of thought and few-shot + CoT prompting techniques that demonstrated noticeable variability (27.19–70.22%, 27.95– 68.66%, respectively), refer to Fig. 5 For GPT-4o, instruction following showed less variability (1.8–46.69%) compared to chain of thought (25.82–84.83%) and few-shot (12.4–54.26%) prompting techniques that showed considerable variability in accuracies, refer to Fig. 6 Gemini showed minimal variability in accuracies for few-shot prompting technique (69.30–73.57%) contrary to instruction following demonstrating noticeable variability (5.16–75.04%), refer to Fig. 7 Claude 3.5 Sonnet showed minimal variability in accuracies for few-shot (36.37– 62.47%) and few-shot + CoT prompting techniques (41.96– 59.32%) compared to high variability demonstrated by instruction following (36.59–77.48%), refer to Fig. 8 Data files that include numerical values relating to Figs. 4, 5, 6, 7, 8 can be found at 10.26187/deakin.30123043.v1, refer to the supplementary appendix as a guide to the files.

**Fig. 9:**
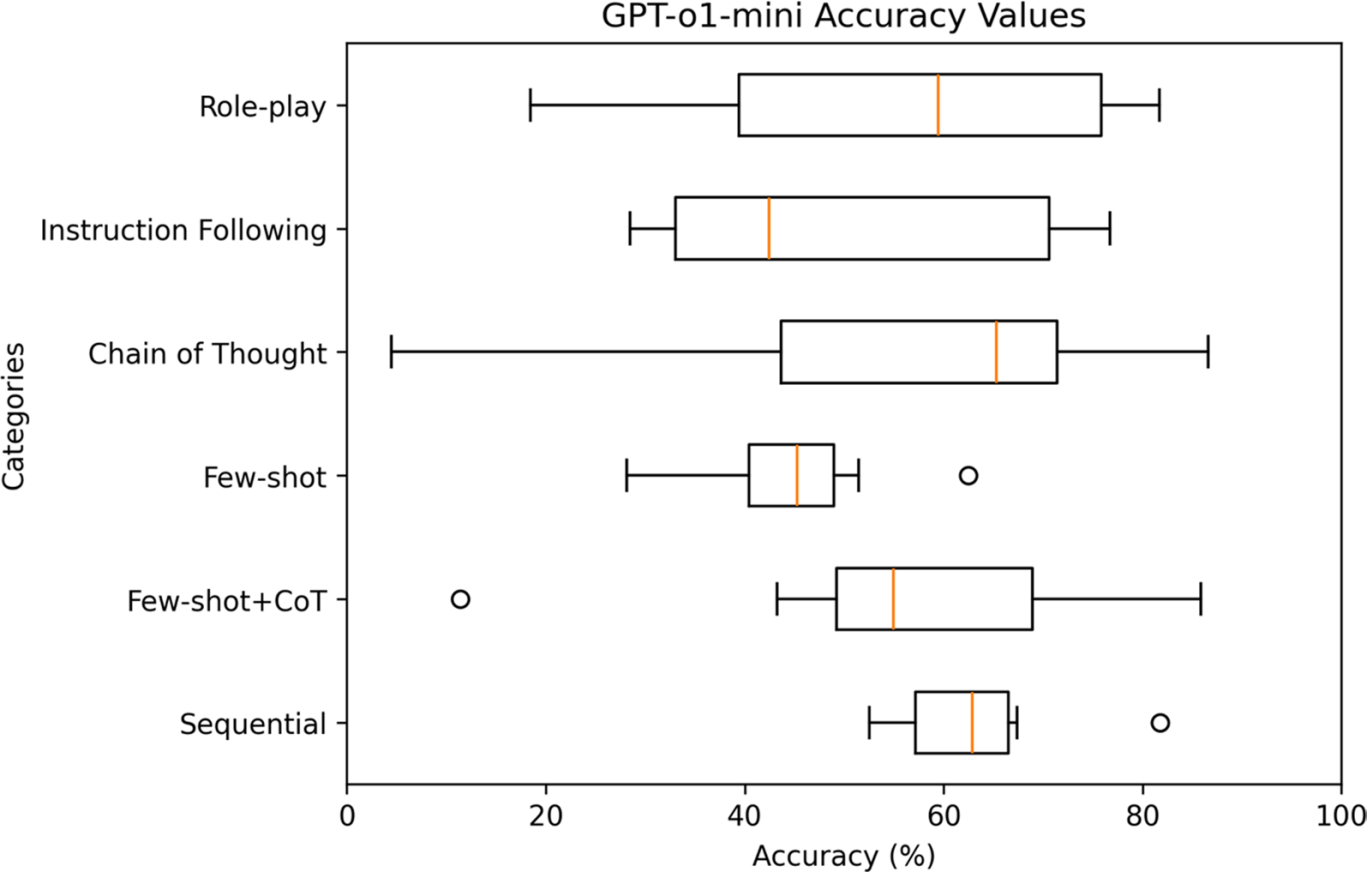
Box plot showing accuracies when six different prompting techniques were executed on GPT-o1-mini model

**Fig. 10:**
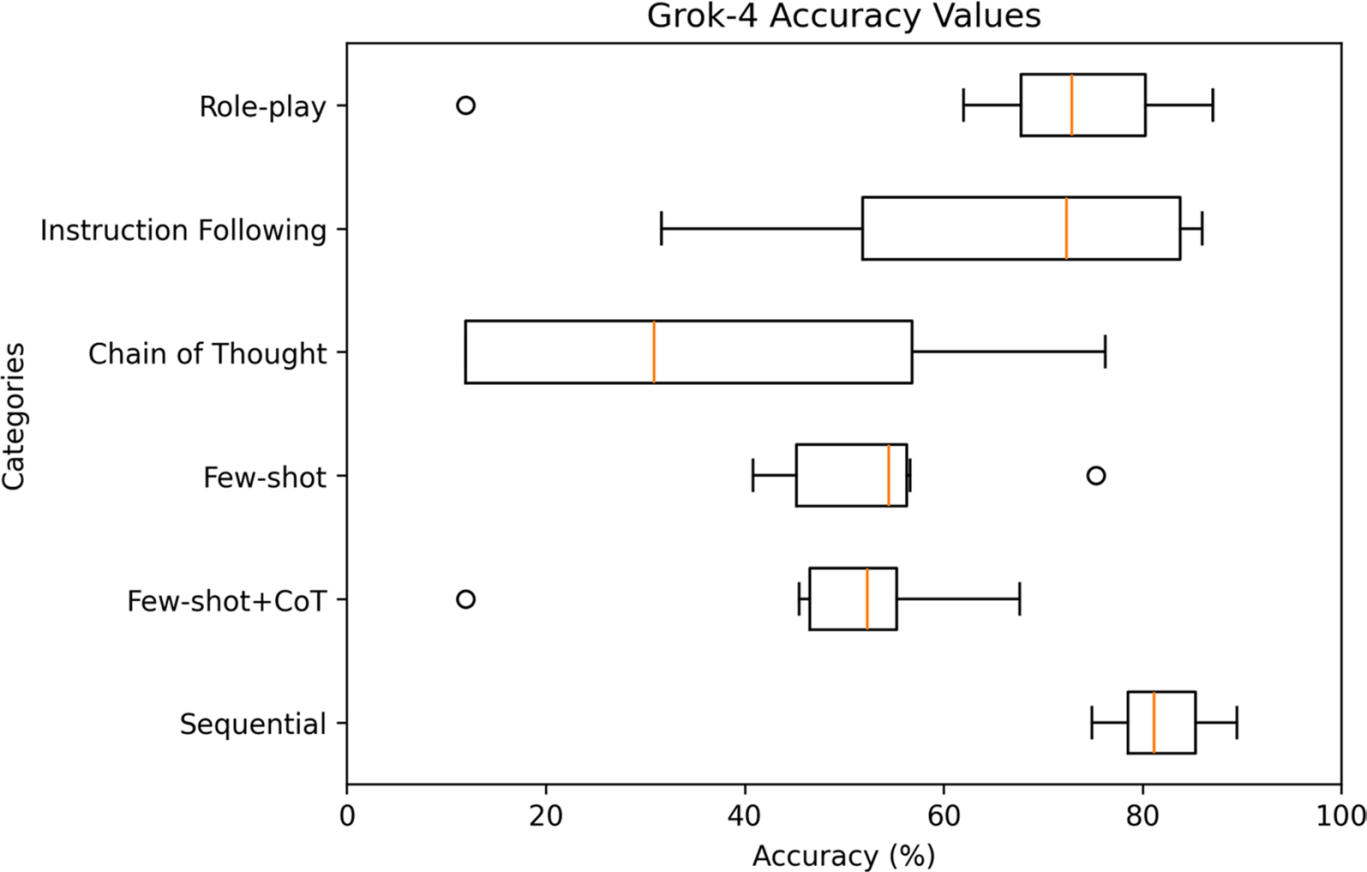
Box plot showing accuracies when six different prompting techniques were executed on Grok-4 model

**Fig. 11:**
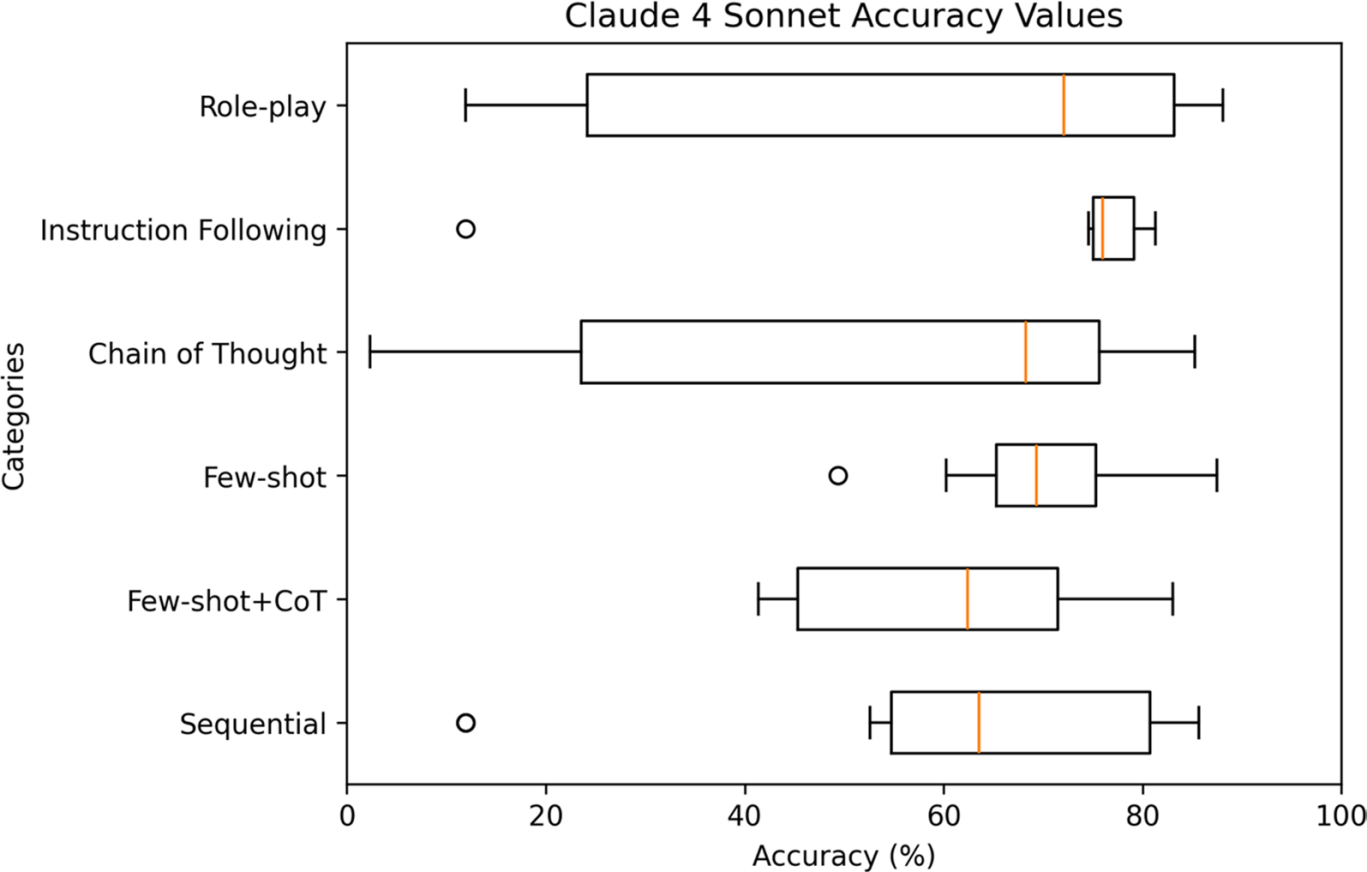
Box plot showing accuracies when six different prompting techniques were executed on Claude 4 Sonnet model

For a comprehensive analysis, Figs. 9–11 highlight the varying levels of accuracy across different prompting techniques on LRMs. For GPT-o1-mini generated rules, sequential with clinical context had minimal impact on the accuracy resulting in low variability (52.55– 81.75%), refer to Fig. 9. In contrast, chain of thought resulted in high variability (4.49–86.57%). For Grok-4, sequential prompting with clinical context showed less variability (74.92–89.47%), compared to instruction following (31.64–85.99%) and chain of thought (11.90–76.26%) prompting techniques that showed considerable variability in accuracies, refer to Fig. 10 Claude 4 Sonnet showed minimal variability in accuracies for few-shot (49.34–87.49%) compared to high variability demonstrated by role-play (11.90–88.07%) and chain of thought (2.30–85.26%) prompting techniques, refer to Fig. 11 Data files that include numerical values relating to Figs. 9–11 can be found at 10.26187/deakin.30123043.v1, refer to the supplementary appendix as a guide to the files.

The interpretability for the LLMs varied between 2 and 25 compared to 41 identified in our PiMS rule set while the interpretability for the LRMs varied between 3 and 94. Tables 6 and 7 summarises the interpretability presenting minimum, maximum, and median values for the LLMs and LRMs respectively when prompted with variables. As shown in Table 6, the distribution of interpretability of LLMs is highly skewed towards low and closer to zero than 41. Additionally, Table 6 shows that while most LLMs had similar median interpretability scores, they were closer to zero than 41 as per the PiMS rule set. However, the distribution of interpretability of LRMs varies across models whereby GPT-o1-mini and Grok-4 is highly skewed towards low compared to 41. Contrary, Claude 4 Sonnet improved interpretability with a median of 27. Our results indicate that the manually created PiMS rule set is more extensive and detailed compared to the LLM and LRM generated rules.

**Table 6:** The minimum, maximum, and median interpretability for the rule sets generated by different large language models compared to an interpretability of 41 for PiMS

Large Language Model	Minimum interpretability	Maximum interpretability	Median interpretability
GPT-3.5	3	11	4
GPT-4	3	14	4
GPT-4o	3	23	4
Gemini	2	14	5
Claude 3.5 Sonnet	3	25	7

**Table 7:** The minimum, maximum, and median interpretability for the rule sets generated by different large reasoning models compared to an interpretability of 41 for PiMS

Large Reasoning Model	Minimum interpretability	Maximum interpretability	Median interpretability
GPT-o1-mini	3	28	9
Grok-4	3	27	6
Claude 4 Sonnet	13	94	27

Our PiMS rule set had a rule complexity of 44. The rule complexity of rules generated by the LLM and LRM varied significantly. The rule complexity of LLMs demonstrated minimum values ranging from 4 to 10 and maximum values spanning from 26 to 71, refer to Table 8 Median rule complexity scores indicate that GPT-4o had the highest value of 25, while Gemini had the lowest value of 14. LRMs resulted in minimum values ranging from 4 to 16 and maximum values ranging from 52 to 183, refer to Table 9 Median rule complexity scores indicate that Claude 4 Sonnet had the highest value of 46, while GPT-o1-mini had the lowest value of 19.

**Table 8:** The minimum, maximum, and median rule complexity for the rule sets generated by different large language models compared to a rule complexity of 44 for PiMS

Large Language Model	Minimum rule complexity	Maximum rule complexity	Median rule complexity
GPT-3.5	5	26	16
GPT-4	8	71	20
GPT-4o	10	46	25
Gemini	4	43	14
Claude 3.5 Sonnet	4	43	20

**Table 9:** The minimum, maximum, and median rule complexity for the rule sets generated by different large reasoning models compared to a rule complexity of 44 for PiMS

Large Reasoning Model	Minimum rule complexity	Maximum rule complexity	Median rule complexity
GPT-o1-mini	4	52	19
Grok-4	14	112	29
Claude 4 Sonnet	16	183	46

Our manual inspection for Part B revealed logical errors. These logical errors included (1) overriding an amber status with a red status and (2) the failure of generated rules assigning uncertain as a system assessment to patient data. This indicates the logical complexity of rule sets, specifically with edge cases. It is evident that a green status (healthy) can be clearly identified; however, for a moderate or severe risk assessment the boundaries are not clearly defined. In addition, it is evident that LLMs do not consider uncertainty. This is evident as it fails to assign uncertainty data that is incomplete (missing data). Our manual inspection of logical errors against the rule complexity of the LLM and LRM generated rule sets is available at 10.26187/deakin.30123043.v1, refer to the supplementary appendix as a guide to the files.

## Discussion

Adopting prompts to LLMs without providing an example or specifying variables resulted in rule sets that were low in comparability to variables in our PiMS rule set. By not specifying variables, there were a range of variables that related to screening and state of emergency regulations including wearing mask, quarantine status, testing capacity, contact tracing, cough duration, breathing difficulty, blood pressure, chest pain, total deaths, number of hospitalizations, curfew violation, total recoveries, social distancing, number of vaccinated people, gathering size in a venue, number of daily cases, hand wash frequency, days since the last exposure, vulnerability, testing capacity, total vaccinations, days since tested positive, PPE demand, and ICU occupancy). However, few-shot prompting included examples resulting in GPT-3.5 and GPT-4 generated rule sets that included variables comparable to PiMS. This highlights the importance of incorporating examples in the prompts. However, the comparison scores were low where GPT-3.5 generated 33% of the PiMS variables and GPT-4, 47% of the variables.

Thus, for Part B we incorporated the variables in the prompts and used LLMs and LRMs. LLMs yielded large variance in average accuracies ranging between 31.62 and 70.71%. Claude 3.5 Sonnet consistently performed well, particularly with chain of thought (66.62%) and instruction following (61.30%) prompting techniques. Gemini had the highest average accuracy using few-shot prompting (70.71%). GPT-3.5 showed strong performance with sequential prompting (65.71%). These results suggest that the prompting techniques have varying impacts on the various LLMs. Less variation in accuracies for few-shot prompting technique for Gemini indicates that the model shows more consistency compared to the other models when examples are provided. However, Gemini’s accuracies dropped significantly for sequential prompting, indicating challenges in handling longer context for example clinical settings. Thus, an LLM achieving these high accuracies underscores the critical role of involving clinicians to obtain keywords (i.e. variables) in prompts during the generative processes.

Average accuracies from LRM varied between 36.28 and 81.70%. LRMs generated rules through sequential prompting with clinical context yielded higher accuracies compared to other prompting techniques. Grok-4 resulted in the highest average accuracy with 81.7%. This highlights the value of structured reasoning and domain grounding in clinical tasks. Grok 4 showed the greatest variance across prompting techniques varying between 36.28 and 81.70% while GPT-o1 and Claude 4 Sonnet demonstrated less variance ranging between 53.38–70.93% and 44.67–62.91%. Chain of thought prompting techniques demonstrated the lowest average accuracy for Grok 4 (36.28%) and Claude Sonnet (53.38%) while achieved the second highest accuracy for GPT-o1 (58.16%). For GPT-o1, few-shot prompting resulted in the lowest average accuracy (44.67%). This suggests that unconstrained reasoning is detriment without contextual understanding. Claude 4 Sonnet showed higher average accuracies with instruction following (70.93%) and few-shot (69.40%) prompting techniques. Overall, sequential prompting with clinical context shows to be the most effective prompting technique indicating that alignment of reasoning steps with domain-specific knowledge is critical.

Interpretability and rule complexity of rule sets generated by LLMs were notably lower compared to those provided by clinicians; where interpretability ranged from (2–25) compared to 41 and rule complexity ranged from 4–71 compared to 44. A low rule complexity for an LLM generated rule set (< 44) indicates that the rule set lacks the essential complexity while a high rule complexity (> 44) implies that the LLMs have introduced accidental complexity that results in logical error. By conducting a manual inspection, we identified that LLMs had “UNCERTAIN” as a default system assessment compared to “AMBER” as a default for PiMS. These logical errors can be reduced specifying a default status in the prompts. For example, explicitly stating to set a default status to “AMBER” and override based on the generated rule sets. Another mitigation strategy is to explicitly define “UNCERTAINTY”. For example, “UNCERTAINTY” is used when there is missing data. These decisions have been predefined by humans and can be used as context to provide background information for LLMs and LRMs. Our results indicate that while LLMs can produce rules that are straightforward and less complex, they lack the nuanced understanding of clinicians.

Interpretability and rule complexity of rule sets generated by LRMs indicates that GPT-o1-mini and Grok-4 generated rule sets with lower median interpretability (9 and 6 respectively), compared to PiMS (41) suggesting limited transparency. In contrast, Claude 4 Sonnet achieved higher maximum interpretability (94) and a median of 27, approaching PiMS. Lower rule complexity of GPT-o1-mini and Grok-4 compared to PiMS suggests that these models support simpler but less complex rule sets, whereas Claude 4 Sonnet can achieve greater interpretability at the cost of increased complexity.

Our methodology of conducting ten iterations for each prompt demonstrated an inconsistency in responses generated by LLMs and LRMs. This emphasises the importance of incorporating repeatability in studies that evaluate LLMs and LRMs regardless of the model or architecture. Therefore, our approach is adaptable and remains relevant regardless of changes or advancements in LLMs and LRMs. The variability in rule sets generated by different LLMs and LRMs highlights the importance of choosing the appropriate LLM or LRM for generating rule sets for CDSSs. However, LLMs and LRMs are continuously being advanced; therefore, leveraging LLMs and LRMs for generating rule sets still requires clinicians for further refinement prior to integration into a CDSS for validation. This research can be extended to improving the LLMs and LRMs through context engineering or agency. By providing context to the LLM, we can increase the accuracy by providing accurate background information enabling AI real world applications. Leveraging AI clinical agency to LLMs and LRMs, we can augment clinicians’ abilities to conduct multiple iterations through iterative feedback for self-refinement while maintaining minimal clinical involvement. We can build agents to facilitate a Delphi Method for consensus commonly practiced in real world clinical scenarios. A moderator agent would send out questionnaires, synthesizes the responses, and provides feedback to the expert agents to encourage consensus. Another technique is supervised fine tuning, given large data sets. These techniques could potentially improve accuracy and completeness of the generated rule set.

## Limitations

We acknowledge that there are several limitations and areas for improvement in our study. We are limited to a single proprietary CDSS. Future work would be to test with different rule sets across various CDSSs including Triaging Systems and Intervention Systems. Based on these systems given there are large data sets, we can use traditional ML techniques as a comparison to LLM generated rule sets. We have considered only six prompting techniques where other prompting techniques such as graph-of-thought, and tree-of-thought were not explored. Open-source LLMs, such as Llama and Falcon, were not assessed, nor were medical domain-specific LLMs such as Med-PaLM. We have considered these prompting techniques and open-source LLMs for future research.

COVID-19 datasets may have been included in the training datasets of LLMs used in this study; therefore, the LLMs might have been biased when generating rules. Gemini and Claude 3.5 Sonnet do not support system prompts; therefore, we incorporated the system prompt into the user prompt when executing experiments for the role-play prompting technique. We compared the LLM generated rules with only one CDSS. Despite LLMs being trained on multiple rule sets, our aim is to evaluate their effectiveness on propriety rule sets. We are currently working with the appropriate governance bodies at public hospitals to obtain additional propriety rule sets. Further, LLM outputs are known to be sensitive to the wording of prompts. Consequently, we had to perform multiple refinements of our prompts before finalizing the prompts used in the study. LRMs used were limited to open-source models and low-cost models.

## Conclusion

The proposed approach demonstrates the potential of generating rules using natural language processing techniques. The results highlight the necessity for further refinement and validation by clinicians (subject-matter experts) to ensure the accuracy of LLM and LRM generated rules before their integration into clinical decision support systems (CDSS). Prompting techniques are essential for the generative processes; therefore, it is important to consider "seeding it with the right context" and the right set of conceptual frameworks to guide the LLM and LRM. Additionally, the complexity and interpretability of the rule sets generated by the LLM using their interpolative structures and internal world models differ from human-crafted representations. The complexity and interpretability of LLM and LRM generated rules being lower compared to those created by clinicians indicating that while LLMs can effectively generate rule sets, they face challenges in creating rules that are comprehensible or practically applicable for clinicians. Despite specifying variables or not in prompts, the rules generated by LLMs and LRMs did not include all the rule sets provided by clinicians. Additionally, LLMs and LRMs have a limited ability to understand and contextualize complex medical data, which can result in incomplete or inaccurate rule generation. Thus, our findings reinforce the necessity of human involvement to generate and review LLM and LRM generated rules that is viable for CDSS. This research can be extended through context engineering or agency. By conducting in-context learning we can provide further background information to guide the LLMs and improve accuracy. Another method is building clinical agency using LLMs and LRMs, we can augment clinicians’ abilities to conduct multiple iterations through iterative feedback for self-refinement while maintaining minimal clinical involvement and conduct agentic Delphi methods to improve accuracy. Another method for improving accuracy would be Supervised Fine Tuning (SFP) and is considered future work that includes further evaluations across various propriety rule sets.

## Supplementary Information

Below is the link to the electronic supplementary material.

Supplementary file 1.

## Data Availability

Data that support the findings of this study are available in supplementary materials. Further data can be requested from the corresponding authors upon request and following institutional review board rules and privacy regulations.
