# Supplementary material for: Large language models and conditional rules in clinical decision support systems
Source: Health Inf Sci Syst. 2026 Jan 21;14(1):32. doi: 10.1007/s13755-026-00428-z (PMC12824032; doi:10.1007/s13755-026-00428-z)
Supplement: Supplementary file 1 [file 13755_2026_428_MOESM1_ESM.docx]

# Supplementary Material

The prompts and outputs associated with our paper, Large language models and conditional rules in clinical decision support systems, are stored in multiple excel files available at https://doi.org/10.26187/deakin.30123043.v1. This supplementary material is a guide for the reader to navigate the files.

### LLM_Generated Rules_Part A

| **Tab** GPT_3.5_Few-shot_Temp1 | |
| --- | --- |
| **Tab** GPT_4_Few-shot_Temp1 | |
| **Columns** | |
| Prompt | Actual prompt submitted |
| Run 1:10 | Output of each run |
| **Rows** | |
| Total number of rule sets | Count of rule sets for each run |
| Domain-specific variables | Variables found in PiMS rule set |
| Accuracy | Accuracy relative to actual PiMS system assessment of each run |
| Rule Complexity | Numerical value for rule complexity of each run |

### LLM_Generated Rules_Part B

| **Tab** LLM_Generated_Rules_1 | |
| --- | --- |
| Temperature | Setting of temperature (0/1) |
| GPT | Transformer Model |
| Prompt Type | Prompting technique |
| Prompt | Actual prompt submitted |
| Output – Run 1:10 | Output of each run |
| Manual Check – Run 1:10 | Outcome of manual check for each run (pass/fail) |
| Accuracy – Run 1:10 | Accuracy relative to actual PiMS system assessment of each run |
| Total number of set rules – Run 1:10 | Count of rule sets for each run |
| Cyclomatic complexity – Run 1:10 | Numerical value for rule complexity of each run |

| **Tab** Accuracy_LLM_1 | |
| --- | --- |
| Model/Prompt | Transformer model and prompt technique |
| Run 1:10 | Accuracy relative to actual PiMS system assessment of each run |
| Avg | Average across the 10 runs |
| Std Dev | Standard deviation for the average |

| **Tab** Interpretability_LLM_1 | |
| --- | --- |
| Model/Prompt | Transformer model and prompt technique |
| Run 1:10 | Count of rule sets for each run |

| **Tab** Rule_Complexity_LLM_1 | |
| --- | --- |
| Model/Prompt | Transformer model and prompt technique |
| Run 1:10 | Numerical value for rule complexity of each run |

| **Tab** Rule_Complexity_Log_Err_1 | |
| --- | --- |
| Rule Complexity | Numerical value for rule complexity of each run |
| Logical Errors Present (Yes/No) | Manual assessment of logical errors against rule complexity |

### LLM_Generated Rules_Part B_Latest Run

| **Tab** LLM_Generated_Rules | |
| --- | --- |
| **Tab** LRM_Generated_Rules | |
| Temperature | Setting of temperature (0/1) |
| GPT | Transformer Model |
| Prompt Type | Prompting technique |
| Prompt | Actual prompt submitted |
| Output – Run 1:10 | Output of each run |
| Manual Check – Run 1:10 | Outcome of manual check for each run (pass/fail) |
| Accuracy – Run 1:10 | Accuracy relative to actual PiMS system assessment of each run |
| Total number of set rules – Run 1:10 | Count of rule sets for each run |
| Cyclomatic complexity – Run 1:10 | Numerical value for rule complexity of each run |

| **Tab** Accuracy_LLM | |
| --- | --- |
| **Tab** Accuracy_LRM | |
| Model/Prompt | Transformer model and prompt technique |
| Run 1:10 | Accuracy relative to actual PiMS system assessment of each run |
| Avg | Average across the 10 runs |
| Std Dev | Standard deviation for the average |

| **Tab** Interpretability_LLM | |
| --- | --- |
| **Tab** Interpretability_LRM | |
| Model/Prompt | Transformer model and prompt technique |
| Run 1:10 | Count of rule sets for each run |

| **Tab** Rule_Complexity_LLM | |
| --- | --- |
| **Tab** Rule_Complexity_LRMs | |
| Model/Prompt | Transformer model and prompt technique |
| Run 1:10 | Numerical value for rule complexity of each run |

| **Tab** Rule_Complexity_Log_Err | |
| --- | --- |
| Rule Complexity | Numerical value for rule complexity of each run |
| Logical Errors Present (Yes/No) | Manual assessment of logical errors against rule complexity |

| **Tab** Null | |
| --- | --- |
| GPT | Transformer Model |
| Prompt Type | Prompting technique |
| Prompt | Actual prompt submitted |
| Output – Run 1:10 | Output of each run |
| Accuracy Without Null values Removed – Run 1:10 | Accuracy relative to actual PiMS system assessment of each run without null values removed |
| Accuracy With Null values Removed – Run 1:10 | Accuracy relative to actual PiMS system assessment of each run with null values removed |

### LLM_Generated Rules

| **Tab** Settings | |
| --- | --- |
| LLM | Transformer Model |
| Temperature | Setting of temperature (0/1) |
| Token | Number of tokens |
| Technique | Prompting technique |
| Prompt | Actual prompt submitted |
| Prompt Token Count | Count of tokens relative to prompt |
| Allowed Token Limit | Token limitations |

| **Tab** GPT_3.5_Few-shot_Temp1 | |
| --- | --- |
| **Tab** GPT_4_Few-shot_Temp1 | |
| **Columns** | |
| Prompt | Actual prompt submitted |
| Run 1:10 | Output of prompts |
| **Rows** | |
| Total number of rule sets – Run 1:10 | Count of rule sets for each run |
| Number of conditions per rule set 1 – Run 1:10 | Count of conditions relative to rule set |
| Number of conditions per rule set 2 – Run 1:10 | Count of conditions relative to rule set |
| Number of conditions per rule set 3 – Run 1:10 | Count of conditions relative to rule set |
| Number of conditions per rule set 4 – Run 1:10 | Count of conditions relative to rule set |
| Average number of conditions per ruleset – Run 1:10 | Average count of conditions relative to rule set |
| Domain-specific variables – Run 1:10 | Relevant variables |
| Accuracy – Run 1:10 | Accuracy relative to actual PiMS system assessment |
| Rule Complexity – Run 1:10 | Numerical value for rule complexity of each run |

| **Tab** GPT_3.5_Few-shot_Temp0 | |
| --- | --- |
| **Tab** GPT_4_Few-shot_Temp0 | |
| Prompt | Actual prompt submitted |
| Run 1:10 | Output of prompts |

| **Tab** GPT_3.5_Responses_Python_Code | |
| --- | --- |
| **Tab** GPT_4_Responses_Python_Code | |
| Technique | Prompting technique |
| Prompt | Actual prompt submitted |
| Round 1:10 Response | Output of prompts |

| **Tab** GPT_3.5_Doc_Prompt_Temp0 | |
| --- | --- |
| **Tab** GPT_4_ Doc_Prompt_Temp0 | |
| Document | Document submitted |
| Prompt | Actual prompt submitted |
| Document content | Relevant content of document |
| Python code | Output of prompts |
